# O02 Persistent non-fulminant COVID-19 infection in a GPA patient on rituximab

**DOI:** 10.1093/rap/rkaa053.001

**Published:** 2020-11-03

**Authors:** Gurpreet Kaur, Alaeldin Nour, Kehinde Sunmboye

**Affiliations:** Rheumatology: University Hospitals of Leicester, Leicester, United Kingdom

## Abstract

**Case report - Introduction:**

Based on initial clinical data, it was suggested that patients with vasculitis who were immunosuppressed, would have a more severe COVID-19 infection. Here we present a case of a young 26-year old lady with granulomatosis with polyangiitis (GPA) on rituximab who developed COVID-19 infection while on active GPA treatment. Her COVID-19 infection confirmed on PCR serology, has been protracted but non-fulminant. She did not require mechanical respiratory support. At the same time her GPA remained active and worsened requiring further immunosuppression after she developed mild pulmonary haemorrhage. She is currently still receiving vasculitis treatment.

**Case report - Case description:**

A 26-year-old lady with a background history of obstructive sleep apnoea and fibromyalgia was diagnosed with ENT-limited GPA in 2017. She was initially treated with azathioprine then methotrexate, and later switched to Rituximab in 2018 after she developed organ-threatening manifestations with bilateral hearing loss. She was stable on periodic infusions of rituximab at 6 to 9-monthly intervals and did not develop other organ-threatening features.

She had been given one dose of rituximab for a flare of her GPA. In between rituximab doses, she was admitted with acute COVID-19 infection with related pneumonia and treated with antibiotics, fluids, and oxygen. Shortly after discharge, she was readmitted with worsening symptoms of non-resolving COVID-19 pneumonia which was evident on chest x-ray and levofloxacin treatment was initiated. Her condition improved and she was discharged. No mechanical respiratory support was required. She had her 2^nd^ dose of rituximab after it had been delayed by about 2 weeks. She had been afebrile after the acute COVID-19 infection and her persistent positive results were explained as related viral shedding over a period of 8 weeks.

One week later, she represented to hospital with fever, cough and shortness of breath, and her blood results showed a remarkable rise in inflammatory markers, including a CRP of 242. She was treated for non-resolving COVID-19 pneumonitis with worsening chest x-ray features. After hospital discharge, her GPA continued to flare with persistent epistaxis with nasal crusting. She also had worsening inflammatory arthritis with purpuric rash on her legs. An ENT review confirmed nasal septum perforation, but no renal involvement was found. Additional cyclophosphamide was commenced via the day-case unit. Her SARS-CoV-2 serology was negative prior to commencing cyclophosphamide. She is now SARS-CoV-2 positive after two doses of cyclophosphamide, but she is afebrile and stable.

**Case report – Discussion:**

COVID-19 infection carries a high mortality rate in patients with multiple co-morbidities, but recent literature suggests that patients on immunosuppressants may not actually have fulminant COVID-19 disease. This case illustrates the challenges of treating active vasculitis in the context of ongoing COVID-19 infection. Her vasculitis remained active requiring escalation of immunosuppression with caution, while she was concomitantly fighting SARS-CoV-2 and superadded bacterial infection. A similar case has been published by Guilpain et al of a 52-year-old woman with PR3-ANCA vasculitis on maintenance therapy with rituximab and low-dose prednisone who developed COVID-19 infection. They reported milder evolution of COVID-19 infection in comparison with previous reports.

It is now well known that some disease-modifying anti-rheumatic drugs (DMARDs) such as tocilizumab, hydroxychloroquine and tofacitinib could suppress the cytokine profile seen in severe COVID-19 infection. In addition, several case reports have even reported possible protective effect of immunosuppressants against severe complications of COVID-19 in patients with rheumatological and non-rheumatological conditions. Another complexity in this case was monitoring the disease progression, since both COVID-19 and GPA can have similar findings on chest CT scan of ground glass opacity. In order to better understand the role of immunosuppressants in rheumatological patients with COVID-19 infection, more data is required, currently European League Against Rheumatism (EULAR) is collecting data to monitor and report outcomes of COVID-19 in adult and paediatric population, this will provide invaluable insight for Rheumatologists.

**Case report - Key learning points**
 

- This case poses a challenge for Rheumatologists in managing a patient with active vasculitis and concomitant COVID-19 infection due to limited data available literature. 
- It has also stressed the importance of working in a multidisciplinary team when managing such complex patients. 
- Importance of continuous surveillance of patients receiving immunosuppressive therapy is advised due to possible increased risk to SARS-CoV-2.

